# MicroRNA-mediated down-regulation of NKG2D ligands contributes to glioma immune escape

**DOI:** 10.18632/oncotarget.2287

**Published:** 2014-07-31

**Authors:** Paula Codo, Michael Weller, Gunter Meister, Emese Szabo, Alexander Steinle, Marietta Wolter, Guido Reifenberger, Patrick Roth

**Affiliations:** ^1^ Laboratory of Molecular Neuro-Oncology, Department of Neurology, University Hospital Zurich, Switzerland; ^2^ Department of Biochemistry I, University of Regensburg, Germany; ^3^ Institute for Molecular Medicine, University of Frankfurt, Germany; ^4^ Department of Neuropathology, Heinrich Heine University Düsseldorf and German Cancer Consortium (DKTK), partner site Essen/Düsseldorf, Germany

**Keywords:** glioma, immune escape, NKG2D, miRNA

## Abstract

Malignant gliomas are intrinsic brain tumors with a dismal prognosis. They are well-adapted to hypoxic conditions and poorly immunogenic. NKG2D is one of the major activating receptors of natural killer (NK) cells and binds to several ligands (NKG2DL).

Here we evaluated the impact of miRNA on the expression of NKG2DL in glioma cells including stem-like glioma cells. Three of the candidate miRNA predicted to target NKG2DL were expressed in various glioma cell lines as well as in glioblastomas in vivo: miR-20a, miR-93 and miR-106b. LNA inhibitor-mediated miRNA silencing up-regulated cell surface NKG2DL expression, which translated into increased susceptibility to NK cell-mediated lysis. This effect was reversed by neutralizing NKG2D antibodies, confirming that enhanced lysis upon miRNA silencing was mediated through the NKG2D system. Hypoxia, a hallmark of glioblastomas in vivo, down-regulated the expression of NKG2DL on glioma cells, associated with reduced susceptibility to NK cell-mediated lysis. This process, however, was not mediated through any of the examined miRNA. Accordingly, both hypoxia and the expression of miRNA targeting NKG2DL may contribute to the immune evasion of glioma cells at the level of the NKG2D recognition pathway. Targeting miRNA may therefore represent a novel approach to increase the immunogenicity of glioblastoma.

## INTRODUCTION

Glioblastomas are intrinsic tumors of the brain with a poor prognosis despite comprehensive therapeutic strategies [1]. They are characterized by diffuse infiltration of the healthy brain, well-adapted to a hypoxic environment and poorly immunogenic, precluding potent anti-tumor immune responses. Overcoming the lack of immunogenicity of glioma cells may help to exploit the immune system as a therapeutic weapon against these tumors. However, this will only be feasible based on a deeper understanding of the underlying mechanisms that preclude active immune responses. Glioma cells can interact and activate immune cells through ligands for activating receptors. One of the most prominent activating NK cell receptors investigated in the context of glioma is NKG2D, a receptor that also provides costimulatory signals to cytotoxic T cells [2]. Engagement of NKG2D initiates a perforin-dependent immune attack [3]. NKG2D recognizes different MHC class I-homologous ligands (NKG2DL), including the MHC class I-chain related molecules A (MICA) and B (MICB) and the UL16-binding proteins (ULBP)1-6 [4, 5], which are also present on the surface of glioma cells [6]. However, the expression of NKG2DL by glioma cells is counteracted by different glioma-derived mechanisms that impede glioma cell recognition by immune cells. The NKG2D system is suppressed by transforming growth factor (TGF)-β, a master immunosuppressive cytokine expressed by glioma cells, by at least three mechanisms: TGF-β (i) down-regulates the expression of NKG2D on immune effector cells, (ii) reduces MICA and ULBP2 expression levels on the surface of glioma cells [7-9], and promotes NKG2DL protein cleavage from the cell surface in a metalloproteinase-dependent manner [8].

microRNAs (miRNA), small non-coding RNA molecules that control numerous cellular mechanisms by inhibition of translation and/or enhanced degradation of specific mRNA, are involved in the regulation of NKG2DL expression in colon and prostate cancer cells as well as melanoma [10-12]. In the context of gliomas and most other tumor entities, altered miRNA expression contributes to the malignant phenotype through deregulation of gene expression at the post-transcriptional level [13]. Here, we characterized the role of miRNA in the regulation of NKG2DL expression in glioma cells as well as their functional impact for the immunogenicity of these tumors.

## RESULTS

### NKG2DL are expressed in LTC and GIC

**Fig.1:**
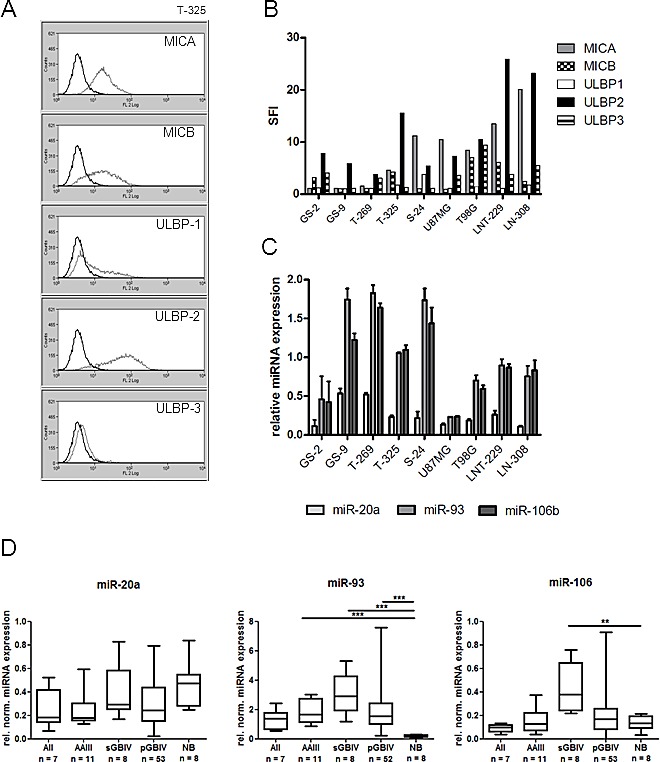
NKG2DL and NKG2DL-regulating miRNA are expressed by LTC and GIC A, B. NKG2DL expression was assessed in a panel of glioblastoma cell lines. The levels of MICA, MICB, ULBP1, ULBP2 and ULBP3 on the cell surface of T-325 cells are shown in A (specific antibody light grey; isotype control dark grey). Specific fluorescence indexes (SFI) are indicated in B. C. miR-20a, miR-93 and miR-106b levels were analyzed by real-time PCR using RNU48 as reference. Relative expression levels are shown. D. The expression of three candidate miRNA in tissue samples of normal brain and gliomas of different WHO grades were assessed using TaqMan^TM^ microfluidic card analyses. Normalized relative expression values are indicated for each tumor group (AII, low-grade astrocytoma, AAIII, anaplastic astrocytoma, pGBIV and sGBIV, primary and secondary glioblastoma) and non-neoplastic brain tissue sample (NB). Boxplots indicate the median expression levels as well as upper 75^th^ percentiles and lower 25^th^ percentiles. Whiskers represent the minimal and maximal expression values, respectively. Statistical comparisons between groups were performed with the Kruskal-Wallis test and Dunn's correction for multiple testing (** p<0.01; *** p<0.001).

We have previously shown that NKG2DL are present on the cell surface of glioma long-term cell lines (LTC) [6]. Here, we assessed the expression of five different NKG2DL on glioma-initiating cell (GIC) lines and confirmed their expression in the LTC lines LNT-229, LN-308, T98G and U87MG. Staining with monoclonal antibodies to MICA, MICB, ULBP1, ULBP2 or ULBP3 and subsequent analysis by flow cytometry (exemplified in Fig. 1A) demonstrated the presence of most NKG2DL on the surface of the tumor cells (Fig. 1B). Overall, higher expression levels were noted in LTC than in GIC.

### Human glioma cells express putative NKG2DL-targeting miRNA *in vitro* and *in vivo*

Based on the description of NKG2DL-regulating miRNA in cancer cell lines as well as *in silico*-based prediction using TargetScan and miRanda (www.targetscan.org; www.microRNA.org; [10, 14]), we selected six miRNA candidates as putative regulators of different NKG2DL in glioma cells. Three of these, miR-20a, miR-93 and miR-106b, were detected in all glioma cell lines assessed, with miR-93 being most abundantly expressed (Fig. 1C). Three other candidate miRNA for NKG2DL regulation, miR-302, miR-372 and miR-373, were investigated but not detected in any cell line examined. We also assessed the expression of all six candidate miRNA *in vivo* using tissue specimens of gliomas of different WHO grades. TaqMan^TM^ Array MicroRNA card analysis confirmed the expression of miR-20a, miR-93 and miR-106b in human gliomas *in vivo* (Fig. 1D). Looking specifically at gliomas of different WHO grade, miR-93 expression levels were higher in any glioma compared to normal brain whereas for miR-20a and miR-106b a mixed expression pattern was observed (Fig. 1D). Consistent with the *in vitro* findings, miR-302, miR-372 and miR-373 were not detected in any glioma tumor sample. Thus, we focused for all subsequent studies on the broadly expressed miR-20a, miR-93 and miR-106b.

### LNA-mediated miRNA silencing up-regulates NKG2DL cell surface expression

**Fig.2:**
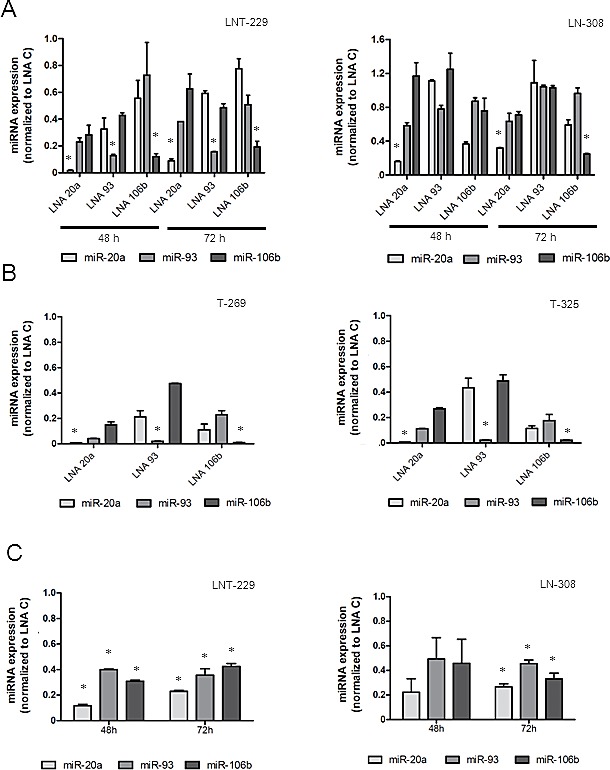
LNA molecules down-regulate miRNA expression in glioma cells A. LNT-229 or LN-308 cells were exposed to LNA molecules specific for single miRNA inhibition or containing a scrambled control sequence. miR-20a, miR-93 and miR-106b expression levels were determined by real-time PCR. B. T-269 and T-325 GIC cells were treated and analyzed as in (A) (* p<0.05). C. LNT-229 or LN-308 cells were exposed to a LNA triple combination (25 nM for each LNA, 75 nM in total). miRNA levels were assessed by real-time PCR.

**Fig.3:**
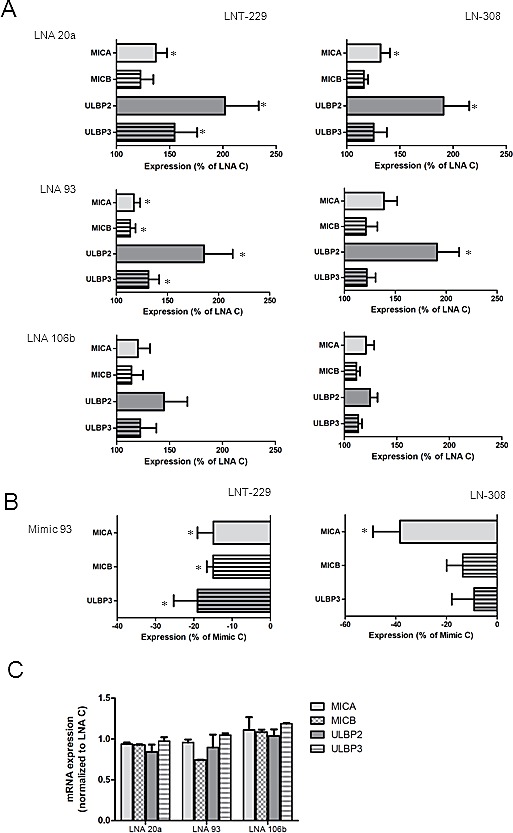

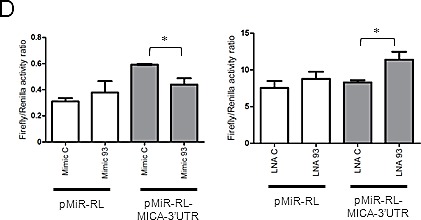
LNA-mediated miRNA inhibition results in increased NKG2DL expression A. LNT-229 or LN-308 glioma cells were exposed to single specific LNAs or scrambled control LNA molecules at 50 nM. The cells were harvested and analyzed after 48 h for NKG2DL cell surface expression by flow cytometry. Expression is shown as % of control for every NKG2DL which displayed an increased expression with at least one LNA inhibitor (* p<0.05). B. LNT-229 cells or LN-308 were transfected with miR-93 mimic or scrambled control at a concentration of 50 nM and NKG2DL expression was analyzed by flow cytometry. Expression is shown as % of reduction respect to control mimic in 4 independent experiments (* p<0.05). C. The cells were treated as in (A) and analyzed for NKG2DL mRNA expression by qPCR after 72 h. D. Direct targeting of the 3’UTR of MICA was analyzed by luciferase reporter constructs using LN-308 cells. The cells were co-transfected with the reporter plasmid and miR-93 mimic, LNA 93 or the corresponding controls for 24 h. Glioma cells transfected with the empty reporter construct (pMiR-RL) were used as control (* p<0.05).

In order to assess the influence of the candidate miRNA on NKG2DL expression, we used LNA inhibitors to silence miR-20a, miR-93 or miR-106b expression in glioma cells. The effect of tumor cell exposure to LNA molecules on miRNA expression levels was evaluated by real-time PCR at different time points. As shown in Fig. 2A, LNA treatment inhibited miRNA expression in LNT-229 and LN-308 cells at 48 h and 72 h after transfection. A similar down-regulation was achieved upon exposure to LNA inhibitors in the GIC lines T-269 and T-325 (Fig. 2B). In general, LNA molecules, regarded as target-specific, had most prominent effects on their target miRNA, however, we also observed cross-inhibition among miR-20a, miR-93 and miR-106b. These effects are likely due to the fact that all 3 miRNA share the same seed sequence (nucleotides 2 to 8). The combination of all 3 LNA inhibitors resulted in a strong down-regulation of all miRNA of interest (Fig. 2C). However, the combination of all 3 LNA inhibitors did not result in a stronger reduction of one of the miRNA candidates compared to treatment with a single specific LNA inhibitor as shown in Fig. 2A. As a next step, glioma cells, exposed to LNA inhibitors were analyzed for the cell-surface expression of NKG2DL at different time-points after transfection using flow cytometry. LNA treatment resulted in an increase of NKG2DL on the cell surface of LNT-229 and LN-308 cells (Fig. 3A). Although showing the same trend as LNA 20 and LNA 93, LNA 106b-induced changes were not statistically significant. The triple combination of LNAs was not more efficient in the up-regulation of NKG2DL than single LNA molecules (data not shown). Furthermore, we detected only minor changes in NKG2DL cell surface levels of GIC lines except for ULBP3, which was elevated upon exposure to LNA 93 in T-269 cells (Suppl. Fig. 1). In line with the findings obtained with LNA inhibitors, treatment of LNT-229 cells with a miR-93 mimic decreased the cell surface expression of MICA, MICB and ULBP3 (Fig. 3B). Similar results were obtained when LN-308 cells were treated with miR-93 mimics. Up-regulation of NKG2DL protein levels upon LNA treatment was not associated with an increase of NKG2DL transcripts suggesting that the observed effect on NKG2DL protein is due to translational repression and not caused by altered mRNA stability (Fig. 3C and data not shown). Next, we confirmed the specific interaction between a candidate miRNA and the 3’UTR of selected NKG2DL. The 3’UTR of MICA was cloned into the pMIR-RL dual luciferase vector. LN-308 glioma cells were co-transfected with reporter plasmid and miR-93 mimic or LNA 93 as indicated in the methods section. Transfection with the miR-93 mimic resulted in decreased reporter activity indicating that miR-93 interacts with the 3’UTR of MICA. Consequently, exposure to LNA 93 increased the activity of the reporter system suggesting an inhibitory activity of the LNA molecules on endogenous miR-93 in LN-308 cells (Fig. 3D). Similarly, exposure to the miR-93 mimic down-regulated MICA 3’UTR reporter activity in LNT-229 and T98G glioma cells (data not shown) corroborating the finding of a direct inhibitory effect of miR-93 on the 3’UTR of MICA.

### NK cell-mediated cytotoxicity in vitro is enhanced by inhibition of NKG2DL-targeting miRNA

**Fig.4:**
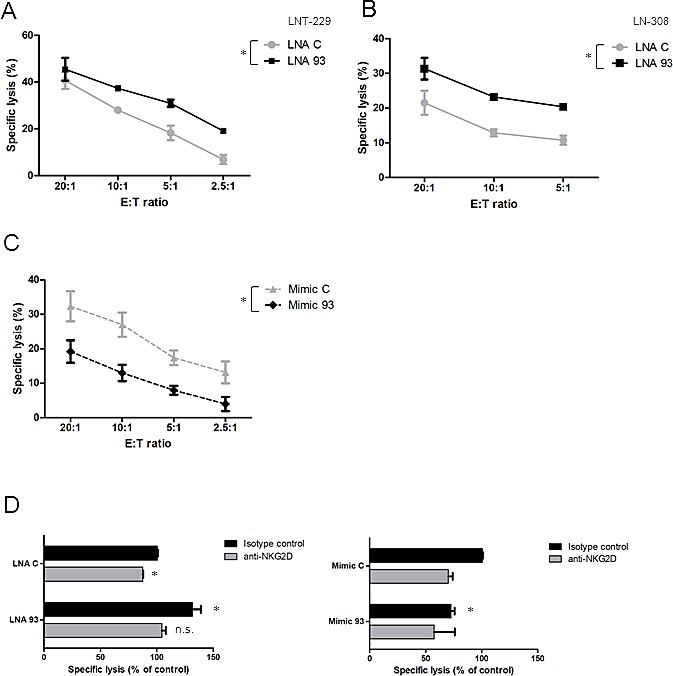
LNA-mediated miRNA inhibition enhances glioma cell susceptibility to immune cell lysis A, B. LAK cells were used in a 3.5 h immune cell lysis assay with LNT-229 (A) or LN-308 cells (B), pretreated with control or LNA 93 molecules for 48 h, as target cells. Data are expressed as specific lysis at different effector:target (E:T) ratios for independent experiments (* p<0.05, ANOVA). C. LNT-229 cells were exposed to miR-93 mimics (mimic-93) or the respective control molecules and subsequently used as target cells in an immune cell killing assay as in (A). D. LAK cells were pre-incubated with anti-NKG2D antibody or isotype control for 1.5 h and subsequently used as effector cells in a killing assay as in (A) against LNT-229 target cells pre-exposed to LNA 93 or miR-93 mimic at an E:T ratio of 20:1. Data are expressed as % of lysis of cells treated with control LNA plus isotype antibody (* p<0.05, n.s.= not significant).

Next we determined the functional role of miRNA that control NKG2DL expression for the immunogenicity of glioma cells. As shown in Fig. 3A, the most prominent effects on NKG2DL expression were obtained after modulation of miR-93 activity. Accordingly, lymphokine-activated killer (LAK) cells were used as effector cells in a killing assay with LNT-229 or LN-308 cells pre-exposed to either control or LNA 93 molecules. Treatment with LNA 93 resulted in significantly increased immune cell-mediated lysis compared to control LNA-treated cells (Fig. 4A and B). To confirm these findings, we treated both cell lines with LNA 20a, which had also resulted in a significant upregulation of NKG2DL (Fig. 3A). Again, an increase in immune cell-mediated cytolysis was observed (Suppl. Fig. 2). The specific modulation of glioma cell immunogenicity was further corroborated by exposure of LNT-229 cells to miR-93 mimics, which resulted in reduced immune cell killing (Fig. 4C). Finally, we determined whether the increased glioma cell immunogenicity depends on the induction of NKG2DL in response to LNA treatment. Pre-incubation of LAK cells with NKG2D blocking antibodies reversed the enhanced susceptibility of LNT-229 glioma cells to immune cell killing upon LNA 93 exposure, indicating that the observed effects are indeed mediated through a modulation of the NKG2D system. Conversely, when NKG2DL expression was reduced by miR-93 mimics, NKG2D blocking antibodies no longer decreased residual lysis (Fig. 4D).

### Hypoxia contributes to glioma immune escape through NKG2DL down-regulation in a miRNA-independent manner

**Fig.5:**
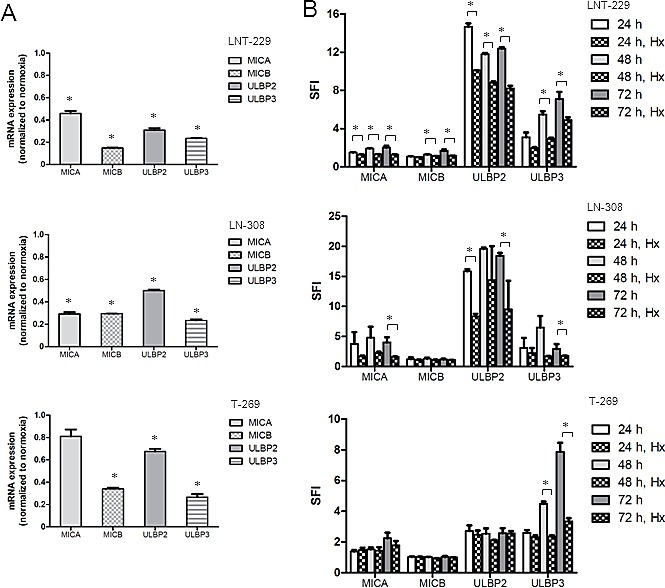

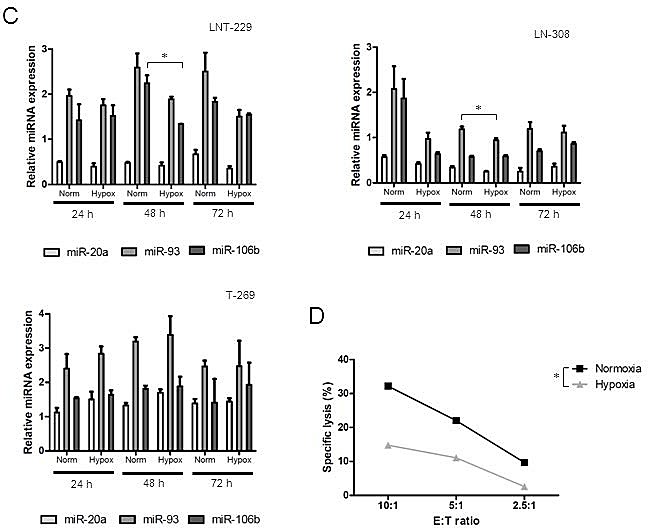
Hypoxia reduces NKG2DL expression on glioma cells in a miRNA-independent manner A. Glioma cells were cultured under normoxic or hypoxic conditions for 24, 48 and 72 h and subsequently assessed for NKG2DL expression by real-time PCR (* p<0.05). B. The cells were cultured under normoxic oder hypoxic (Hx) conditions as indicated and assessed for NKG2DL levels at the cell surface by flow cytometry (* p<0.05). C. The cells were treated as in (B) and miRNA expression levels were determined by real-time PCR using RNU48 as a control (* p<0.05). D. LNT-229 glioma cells were cultured under hypoxia or normoxia for 48 h and subsequently used as targets in a LAK cell lysis assay. Data are expressed as specific lysis at different E:T ratios from independent experiments (* p<0.05, ANOVA).

Hypoxia is a hallmark of high-grade gliomas and has been proposed to support glioma-induced immunosuppression [15]. We therefore tested whether hypoxia modulates NKG2DL expression and whether such an effect is mediated by altered miRNA expression. LNT-229, LN-308 or T-269 cells were cultured under normal or hypoxic conditions for 24, 48 or 72 h. Up-regulation of CAIX confirmed that cells became hypoxic (Suppl. Fig. 3). We observed a sustained hypoxia-dependent down-regulation of MICA, MICB, ULBP2 and ULBP3 transcripts in all 3 cell lines (Fig. 5A). Down-regulation on the protein level at the cell surface was confirmed by flow cytometry (Fig. 5B). However, this decrease in NKG2DL expression was not accompanied by increased miR-20a, miR-93 or miR-106b expression (Fig. 5C). In contrast, some miRNA were even down-regulated in hypoxia suggesting that hypoxia-induced repression of NKG2DL levels is not mediated through increased expression levels of the three candidate miRNA. When LNT-229 cells were cultured under hypoxic conditions for 48 h, they became significantly more resistant to immune cell lysis than cells that had been cultured under standard conditions (Fig. 5D). A similar, albeit less pronounced effect, was found for LN-308 cells (data not shown), highlighting the contribution of hypoxia to the immune evasion of glioma cells. Pre-incubation of effector cells with anti-NKG2D blocking antibody resulted in a reduction of specific lysis to a similar extent than the effect observed for target cells that were kept under hypoxic conditions before lysis. NKG2D blocking antibodies further decreased the sensitivity of hypoxic target cells to immune cell killing, albeit to a lesser extent than in normoxic conditions reflecting the activity of residual NKG2DL expression in hypoxia (Suppl. Fig. 4).

## DISCUSSION

The treatment of glioblastoma has been challenging and many therapeutic approaches have largely failed including the administration of anti-angiogenic drugs, which do not prolong overall survival [16, 17]. Therefore, there is a continuous interest in defining novel molecular targets and strategies that may results in more encouraging results. The brain has been called an ‘immunoprivileged site’ because of several factors such as the presence of the blood-brain barrier and the lack of professional antigen-presenting cells that hamper immune responses. However, as can be observed in some pathological conditions such as inflammatory diseases, powerful immunological reactions occur in the central nervous system. Based on these considerations, therapeutic approaches aiming at exploiting the immune system against brain tumors hold promise and have gained increasing interest within the last years. However, malignant brain tumors and foremost glioblastoma are characterized by a variety of tumor-derived mechanisms contributing to their immune escape [18]. Overcoming the lack of immunogenicity is a prerequisite for effective anti-tumor immune responses. Here, we characterized the regulation of the expression of ligands to one of the most prominent activating immune cell receptors, that is, NKG2D. Overall, constitutive NKG2DL expression by glioma cells is too low to efficiently promote immune cell activation *in vivo* [6]. We speculated that miRNA contribute to the down-regulation of NKG2DL expression on glioma cells. First, we identified the expression of three candidate miRNA, miR-20a, miR-93 and miR-106b, pre-selected by literature search as well as TargetScan and miRanda interrogation [10, 14], which may regulate NKG2DL expression (Fig. 1C). In contrast, three additional candidate miRNA were not detected in any cell line. These findings were corroborated by an analysis of fresh-frozen glioma tissue, which also revealed the expression of the first three candidates but not of the other three miRNA (Fig. 1D), confirming the reliability of the findings in *in vitro* cultured cell lines. We next sought to determine the functional impact of the three candidate miRNA on NKG2DL levels on the surface of glioma cells. To this end, we used LNA molecules to antagonize the function of single miRNA candiates which resulted in a strong down-regulation of the respective miRNA. However, we also noticed overlapping effects of the LNA molecules on the other two miRNA, which can be explained by the seed sequence which is shared by all 3 miRNA candidates (Fig. 2A-C). Flow cytometric analysis of glioma cells exposed to LNA revealed enhanced surface expression of NKG2DL (Fig. 3A). In line with these findings, treatment of glioma cells with miR-93 mimic decreased the expression of MICA and MICB (Fig. 3B). The specific interaction between endogenous miR-93 and the 3’UTR of MICA was demonstrated with an appropriate reporter assay system (Fig. 3D). However, since miRNA typically target numerous gene transcripts, the observed NKG2DL regulation may not only originate from direct interaction with the 3’UTR but also result from posttranscriptional and posttranslational mechanisms which are influenced by the examined miRNA. In summary, our findings indicate that NKG2DL are indeed controlled by miRNA in glioma cells, a mechanism which has also been described for other tumor cells [10-12]. Only minor changes in NKG2DL cell surface expression of GIC were detected upon LNA treatment, suggesting that NKG2DL levels are regulated rather on a transcriptional or posttranslational level in these cultures [7, 8], but not by the miRNA examined here. The upregulation of NKG2DL protein levels upon LNA treatment in LTC was not associated with an increase of NKG2DL transcripts suggesting that the observed effect on NKG2DL protein is due to a translational repression and not caused by altered mRNA stability. Most importantly, we confirmed the functional importance of miRNA-dependent regulation of NKG2DL in glioma cells using immune cell lysis assays (Fig. 4A-C). When the NKG2D system was inhibited with specific antibodies, the LNA-mediated increase in immune cell lysis was abrogated, indicating that the observed effects are indeed mediated through a miRNA-dependent modulation of the NKG2D system (Fig. 4D). These findings corroborate the functional impact of the NKG2D system for the interaction between glioma cells and the immune system [6, 9]. Therapeutic strategies aiming at exploiting NK cells as effector cells against gliomas have shown promising results in preclinical studies [19, 20]. Our results provide additional evidence for the importance of NK cells for tumor surveillance and support the further investigation of NK cell-based strategies against these tumors [19, 21-24].

Finally, we aimed at determining the influence of hypoxia, a hallmark of glioblastoma, on the set of miRNA examined here. miRNA expression can be induced by hypoxia [25] and hypoxia contributes to the immune escape of gliomas in a *signal transducer and activator of transcription 3* (STAT3)-dependent manner via induction of regulatory T cells [15]. Our experiments reveal hypoxia-mediated down-regulation of NKG2DL expression as an additional mechanism which contributes to the immune evasion of these tumors (Fig. 5). An effect of hypoxia on MICA levels had been shown before in prostate and osteosarcoma cell lines [26, 27]. Our experiments demonstrate a pronounced effect of hypoxia on different NKG2DL and reduced immunogenicity as a functional consequence. However, hypoxia-dependent NKG2DL regulation was not mediated through any of the miRNA examined in our cell lines.

In summary, our study highlights the contribution of miRNA to the impaired immunogenicity of glioma cells. Given the rapidly evolving techniques which allow for an *in vivo* targeting of miRNA [28], inhibition of the miRNA described in this project may be exploited as an immunotherapeutic strategy against glioblastoma.

## MATERIALS AND METHODS

### Cells and reagents

The human glioma long-term cell lines LN-18, LNT-229 and LN-308 were kindly provided by N. de Tribolet (Lausanne, Switzerland). U87MG and T98G glioma cells were purchased from the American Type Culture Collection. The GIC sphere cultures S-24, T-269 and T-325 were generated as previously described [29]. The GS-2 and GS-9 GIC lines were kindly provided by K. Lamszus [30]. All cell lines were authentified by DNA typing at the Leibniz-Institut DSMZ GmbH (Braunschweig, Germany). LTC growing as adherent phenotype were maintained in Dulbecco's modified eagle medium (DMEM, Invitrogen, Life Technologies, Carlsbad, CA), containing 10% fetal calf serum (FCS) (VWR Lonza, Leighton Buzzard, UK) and supplemented with 2 mM glutamine (Invitrogen, Life Technologies), in a 5% CO_2_ incubator at 37°C. GIC were maintained as sphere cultures in Neurobasal A medium (Invitrogen, Life Technologies) supplemented with EGF 10 ng/ml, FGF 10 ng/ml (Peprotech, Rocky Hill, NJ), Heparin 31.5 U/ml (Sigma Aldrich, St. Louis, MO), 1% Glutamax (Invitrogen, Life Technologies) and 2% B27 (Invitrogen, Life Technologies). Cells were detached with Accutase (Gibco, Life Technologies, Carlsbad, CA). When hypoxic conditions were needed, cells were kept in a hypoxia chamber adjusted to 5% CO_2_ and 1% O_2_ at 37°C.

Buffy coats (Blutspende Zurich, Switzerland) were used for the generation of LAK cells. For the selection of CD56+ NK cells, appropriate beads for magnetic cell sorting (MACS, Miltenyi Biotech, Bergisch Gladbach, Germany) were used. Immune cells were kept in RPMI 1640 (Gibco, Life Technologies) supplemented with 9% FCS, 2 mM glutamine (Gibco, Life Technologies), and 1,000 U/ml of recombinant human interleukin (IL)-2 (Peprotech) in 5% CO_2_ atmosphere and 37°C.

### MicroRNA expression analyses in primary glioma tissue samples

Expression of the six selected candidate miRNA was investigated in primary glioma tissue samples from 79 patients, including seven patients with diffuse astrocytoma, World Health Organization (WHO) grade II (AII), 11 patients with anaplastic astrocytoma, WHO grade III (AAIII), eight patients with secondary glioblastoma, WHO grade IV (sGBIV) and 53 patients with primary glioblastomas, WHO grade IV (pGBIV). Investigation of these tumor samples was approved by the institutional review board of the Medical Faculty at Heinrich Heine University Düsseldorf (study number 3482). Total RNA was extracted from fresh-frozen tumor samples using ultracentrifugation as described [31]. Only tissue samples with a histologically estimated tumor cell content of 80% or more were used for RNA extraction. In addition to the tumor tissue specimens, we investigated RNA samples from eight non-neoplastic control brain tissues. These were obtained from commercial sources (#540005 from Stratagene, Cedar Creek, TX; #AM7962 from Ambion, Huntington, UK; #R1234051-50, #R1234035-50, #R1234062-50, #R1234042-10, #R1234045-10, and #R1234078-50 from Biochain, Newark, CA). Analyses for miRNA expression were performed using the TaqMan^TM^ PCR-based miRNA microfluidic card system from Applied Biosystems (Life Technologies) according to the manufacturer's protocol. Expression values of each of the investigated candidate miRNA were normalized against four reference miRNA (miR-30a-5p, miR-30b, miR-30c, and miR-30d), which showed the most stable expression over all analyzed samples.

### Transient transfection

Glioma cells were seeded at appropriate density for each cell line in 6-well plates or 6 cm tissue culture dishes (TPP, Trasadingen, Switzerland), allowed to attach overnight and transfected with 50 nM of LNA^TM^ (Exiqon, Vedbaek, Denmark) or MiRidian miRNA mimics (Dharmacon/Thermo Scientific, Lafayette, CO) specific for a certain miRNA. Metafectene Pro (Biontex, Martinsried, Germany) was used as a transfection reagent. To exclude any unspecific effects exerted by the transfection procedure, all experiments including LNA inhibitors or miRNA mimics were performed with scrambled LNA or mimic molecules, which had no effect on the constitutive miRNA expression, as a control. Subsequently, 24 h after the start of transfection, the medium was changed and the cells were kept in experimental conditions for different times as indicated.

### Real-time polymerase chain reaction (PCR)

Total RNA was prepared using the NucleoSpin® kit (Macherey Nagel GmbH, Düren, Germany). Complementary DNA was prepared using the iScript® kit (BioRad, Hercules, CA). For real-time PCR, gene expression was measured in an Applied Biosystems 7300 Real-Time PCR System using the ABI Prism 7000 Sequence Detection System (Applied Biosystems, Carlsbad, CA) with SYBR Green ROX Mix (Thermo Scientific) and primers (Microsynth AG, Balgach, Switzerland) at a final concentration of 0.4 μM.

GAPDH primers have been described by Carraro et al. [32], and carbonic anhydrase IX (CAIX) by Nytko et al. [33]. Other primer sequences were MICA fwd: 5’-CCTTGGCCATGAACGTCAGG-3’, rev: 5’-CCTCTGAGGCCTCGCTGCG-3’; MICB fwd: 5’-ACCTTGGCTATGAACGTCACA-3’, rev: 5’-CCCTCTGAGACCTCGCTGCA-3’; ULBP2 fwd: 5’-TTACTTCTCAATGGGAGACTGT-3’, rev: 5’-TGTGCCTGAGGACATGGCGA-3’; ULBP3 fwd: 5’-CCTGATGCACAGGAAGAAGAG-3’, rev: 5’-TATGGCTTTGGGTTGAGC TAAG-3’.

Total miRNA was prepared using the miRNeasy Mini Kit (Qiagen, Venlo, The Netherlands). Complementary DNA transcription from miRNA and subsequent real-time PCR-FAM detection was performed using specific TaqMan® probes (Applied Biosystems). The general conditions were 40 cycles at 95°C/15 s and 60°C/1 min. Relative quantification of gene expression was determined by comparison of threshold values. All results were normalized to GAPDH for mRNA and to RNU48 for miRNA, and calculated with the ΔCT method for relative quantification [34].

### Flow cytometry

For the examination of NKG2DL cell surface levels by flow cytometry, the following mouse monoclonal antibodies were used at a final concentration of 10 μg/ml: AMO1 (MICA), BMO1 (MICB), AUMO1 (ULBP1), BUMO1 (ULBP2) and CUMO3 (ULBP3) [35]. Mouse monoclonal IgG1-κ (Sigma-Aldrich) was used as isotype control. Following incubation with the primary antibody, a polyclonal goat anti-mouse immunoglobulins/RPE (DakoCytomation, Forth Collins, CO, 1:50) was applied. For the exclusion of dead cells, staining with TO-PRO-3 iodide (642/661) (Life Technologies) (0.1 μM) was performed right before acquisition of samples. Fluorescence was detected in a CyanADP flow cytometer (Beckman Coulter, Nyon, Switzerland). Specific fluorescence indexes (SFI) were calculated by dividing median fluorescence obtained with specific antibody by median fluorescence obtained with control antibody.

### Immune cell lysis assay

Target cells were detached and labeled using the PKH26 Red Fluorescent Cell Linker Kit for general cell membrane staining (Sigma-Aldrich). Incubation with effector cells was performed in a final volume of 550 μl of complete RPMI, at 37°C for 1.5 h in case of LNT-229 and 3.5 h in case of LN-308. Various effector:target (E:T) ratios were used as indicated. In order to block NKG2D, immune effector cells were pre-incubated with mouse anti-human NKG2D LEAF purified antibody (Biolegend, San Diego, CA) for 1.5 h (0.5 μg/ml), and during the lysis incubation time. Effector cells were used when the viability was > 90% on Vicell counting (Beckman Coulter). Dead cell staining was performed right before acquisition of samples with 0.1 μM TO-PRO-3 iodide.

### 3’UTR luciferase gene reporter assay

3’UTR from MICA was cloned into the pMIR-RL dual luciferase vector [36]. Restriction enzymes used for cloning were SpeI and PmeI (New England Biolabs, Ipswich, MA). The sequence of the primers (Microsynth AG) used for cloning was: fwd: 5’-ACTAGTGCAGCTGGGATTCAATT-3’, rev: 5’-GTTTAAACACGCCTCATATCTAC-3’; Dual luciferase assays were performed using 10,000 cells per well in a 96-well plate. Following attachment overnight, the cells were co-transfected with 20 ng of the respective reporter construct and 50 nM of LNA molecules or miRNA mimics. Firefly luciferase activity was normalized to constitutive renilla luciferase activity.

### Statistics

Data are expressed as mean and standard error of the mean. The experiments shown were repeated three times with similar results. Analysis of significance was performed using the one sample t-test for data expressed as %, Student's t-test for miRNA expression analysis and two way ANOVA for lysis assays (GraphPad Prism 5, La Jolla, CA) (* p<0.05). Statistical evaluation of miRNA expression in different groups of astrocytic gliomas and non-neoplastic brain tissues was performed using the Kruskal-Wallis test with Dunn's correction for multiple testing.

## SUPPLEMENTARY MATERIAL FIGURES


