# Supplementary material for: MicroRNA-mediated down-regulation of NKG2D ligands contributes to glioma immune escape
Source: Oncotarget. 2014 Jul 31;5(17):7651–62. doi: 10.18632/oncotarget.2287 (PMC4202151; doi:10.18632/oncotarget.2287)
Supplement: Supplementary file 1 [file oncotarget-05-7651-s001.pdf]

MicroRNA-mediated down-regulation of NKG2D ligands contributes to glioma immune escape

Supplementary Material

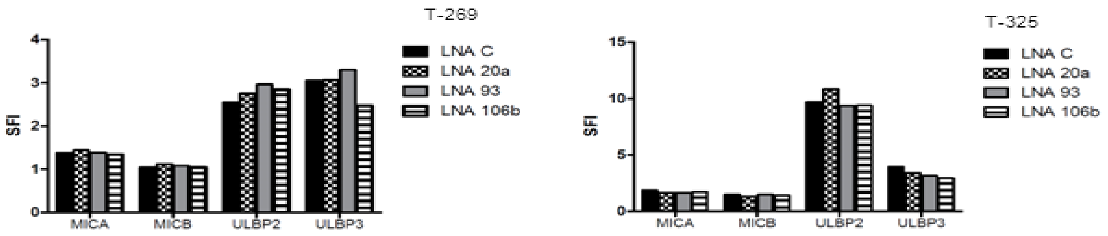

Supplementary Fig. 1: **LNA-mediated miRNA inhibition does not affect NKG2DL expression in GIC.** T-269 or T-325 glioma cells were exposed to single LNAs at 50 nM. The cells were harvested and analyzed after 48 h for NKG2DL cell surface expression by flow cytometry.

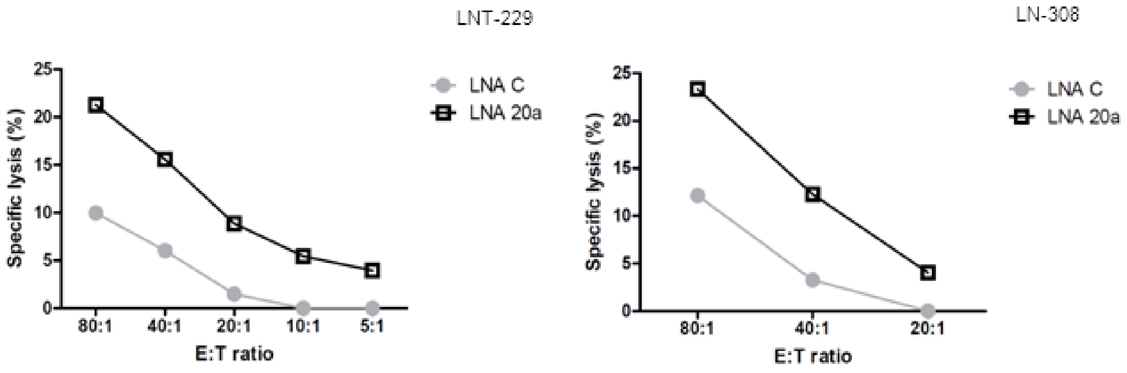

Supplementary Fig. 2: **LNA-mediated miRNA inhibition enhances glioma cell susceptibility to immune cell lysis.** LAK cells were used in a 3.5 h immune cell lysis assay with LNT-229 or LN-308 cells, pretreated with control or LNA 20a molecules for 48 h, as target cells. Data are expressed as specific lysis at different effector:target (E:T) ratios.

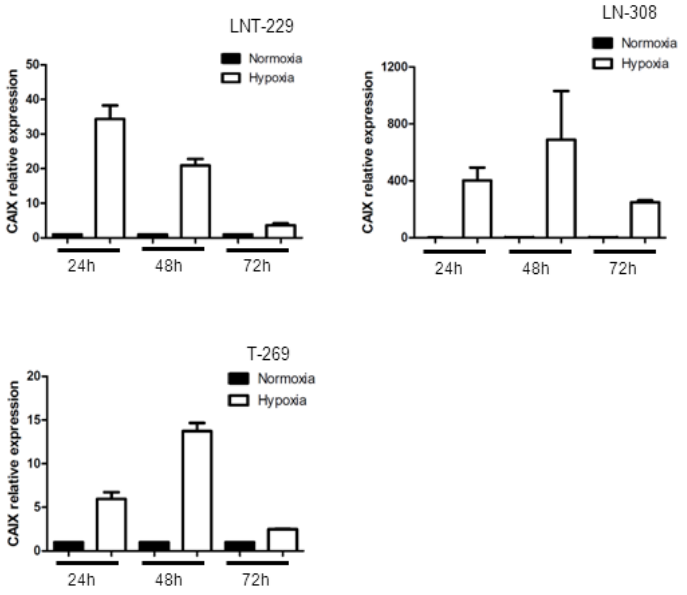

Supplementary Fig. 3: Confirmation of hypoxic conditions by CAIX expression. Glioma cells were cultured under normoxic or hypoxic conditions as indicated and subsequently assessed for CAIX mRNA expression by qPCR. Values are normalized to the respective control incubated in standard oxygen concentration.

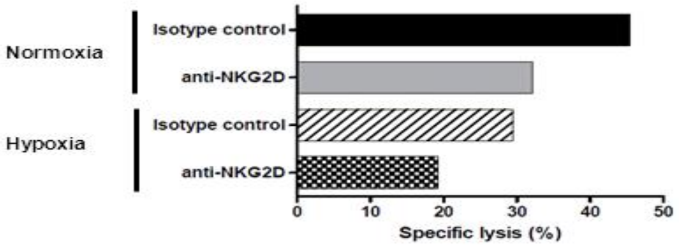

Supplementary Fig. 4: **Reduced immune cell lysis of hypoxic targets is partially mediated through NKG2D signaling.** LAK cells were pre-incubated with anti-NKG2D antibody or isotype control before their use as effector cells in a killing assay at an E:T ratio of 20:1. LNT-229 target cells were cultured under standard oxygen conditions or in hypoxia (1%) for 48 h prior to the experiment.
